# The Impact of Obesity on Breast Cancer Diagnosis and Treatment

**DOI:** 10.1007/s11912-019-0787-1

**Published:** 2019-03-27

**Authors:** Kyuwan Lee, Laura Kruper, Christina M. Dieli-Conwright, Joanne E. Mortimer

**Affiliations:** 1 Division of Biokinesiology and Physical Therapy, University of Southern California (USC), 1540 E. Alcazar Street, CHP 155, Los Angeles, CA 90033 USA; 2 Department of Surgical Oncology, City of Hope Comprehensive Cancer Center, 1500 E Duarte Rd, Duarte, CA 91010 USA; 3 Department of Medical Oncology & Experimental Therapeutics, City of Hope Comprehensive Cancer, 1500 E Duarte Rd, Duarte, CA 91010 USA; 4 Department of Medical Oncology & Experimental Therapeutics, City of Hope National Medical Center, 1500 E Duarte Rd, Duarte, CA 91010 USA

**Keywords:** Obesity, Breast cancer, Diagnosis, Treatment, Clinical outcomes

## Abstract

**Purpose of Review:**

Obesity is a recognized risk factor for the development of breast cancer and recurrence even when patients are treated appropriately. We reviewed the literature that addresses the impact of obesity on diagnosis and the individual therapeutic interventions, and present a summary of the findings.

**Recent Findings:**

Compared to non-obese women with breast cancer, obese women with breast cancer have a worse disease-free and overall survival despite appropriate local and systemic therapies. In brief, obese breast cancer patients experience more complications related to surgery, radiation, and chemotherapy. Further, obese patients are at increased risk for local recurrence compared to normal-weight women. Similarly, systemic chemotherapy is less effective, even when dosed appropriately on the basis of actual weight. Overall, endocrine therapy is less effective in obese women, and there is a suggestion that aromatase inhibitors may be selectively less effective than tamoxifen. Obese women are less likely to undergo breast reconstruction than normal-weight women, and those who do have surgery experience more surgical complications.

**Summary:**

The efficacy of cancer treatments is significantly lower in obese breast cancer survivors, posing greater challenges in patient care and disease management in this patient population. Further investigations are warranted to assess the effects on treatment outcomes and optimize therapeutic mechanisms in order to successfully target breast cancer associated with obesity.

## Introduction

We are in the midst of an obesity epidemic occurring in a society with an increasing incidence of breast cancer. A better understanding of the relationship between obesity and breast cancer is critical because there are unique diagnostic and treatment challenges in this patient population. Obesity was first reported to have an impact on breast cancer diagnosis and outcome in 1976 when Abe et al. reported that obese breast cancer patients, defined as > 20% body weight over their standard weight (height in cm − 100) × 0.9, had larger primary tumors, higher rates of lymphatic invasion, and worse overall survival compared to normal-weight patients [1]. Obesity is associated with worse breast cancer-specific survival [2]. A recent meta-analysis noted that obese women with breast cancer experience up to an 11% decrease in overall survival, regardless of menopausal status [2]. Further complicating survival outcomes, obesity is an established risk factor for metabolic syndrome, type 2 diabetes, and cardiovascular events [3], with the latter continuing to be the main cause of mortality in women with early-stage breast cancer [4]. The reasons for the adverse effects of obesity on breast cancer are numerous and complex. The purpose of this review is to provide an overview of the physical, psychosocial, and biological factors that contribute to a higher incidence of breast cancer and worse clinical outcomes in the overweight and obese patient population.

## Breast Cancer Diagnosis

Obesity impacts the breast cancer trajectory as early as diagnosis. In general, overweight and obese women have poorer compliance with healthy habits and are less likely to comply with screening recommendations such as mammography. A meta-analysis of 16 studies addressed the relationship between body mass index (BMI) and mammography in women aged > 40 and found that overweight women were less likely than normal-weight women to have had a mammogram in the prior 2 years [5]. This was especially true in women with the highest BMI and in Caucasian women, but not in African American women. In looking at barriers to screening to mammography in women aged 50–69, a Kaiser Permanente study found that non-compliance was higher in obese women and that they were twice as likely to cite pain with the procedure as a reason for non-compliance [6].

One might hypothesize that obese women have more fatty tissue in their breasts and that this should decrease breast density, making the mammographic identification of a cancer more obvious. In contrast, it has been shown that the sensitivity of mammography is similar in obese and non-obese women. Older series reported that false-positive findings were 20% higher in obese women compared to normal- or low-weight women [7]. However, a prospective study using mammography registries from seven states conducted on 287,115 postmenopausal women who were not on hormone replacement therapy addressed the impact of screening mammography on breast cancer incidence. Between 1996 and 2005, 614,562 exams were performed and 4446 women were diagnosed with breast cancer. The incidence of hormone receptor (HR)-positive, but not HR-negative, breast cancer increased with increasing BMI. While the false-negative rate was comparable among women with the entire range of BMIs, screen-detected cancers were identified more often and at a more advanced stage in obese women [8]. From this well-designed trial, it was concluded that neither patterns of use nor mammographic accuracy contributed to the increased incidence in obese women. Their findings were further reinforced by the publication of a more recent series. Taken together, the data suggest that obesity is an independent risk for breast cancer [9•].

## Patient and Physician-Related Barriers

Psychosocial factors contribute to the challenge of diagnosing and treating obese women with breast cancer. In general, women of lower socioeconomic status are more likely to be obese than women of higher socioeconomic status and this may complicate access to medical care [10]. The stigma of obesity results in fear, fatalism, alienation, low self-esteem, and embarrassment, all of which contribute to lower adherence to screening and treatment guidelines [11–13]. Friedman et al. qualitatively examined the reasons why some obese breast cancer patients undergo cancer screening. In-depth interviews of 33 women with a mean weight of 263 pounds (sd 45) were recorded for 60–90 min [14]. Friedman et al. verified that obese breast cancer patients share many of the barriers previously identified in the general population including embarrassment, fear of pain, fear of cancer, and competing demands on their time, suggesting that these barriers are not unique to obese breast cancer survivors. A major finding in this study was that an individual’s personality is an important mediator of health behavior. Those participants who followed through on cancer screenings shared certain personality traits compared with unscreened women, such as conscientiousness or self-regulatory ability, which allowed them to complete difficult or feared tasks. The attitudes of healthcare providers may also be a contributing factor in the creation of barriers to healthcare among obese patients. All too frequently, healthcare providers may reflect negative societal attitudes toward obese individuals and this may impact perceptions, judgments, and decision making [15].

## Surgery

### Lumpectomy and Radiation

Patients who are obese are at increased risk for complications with anesthesia as they may be more difficult to intubate and maintain ventilatory support than normal-weight women. An increase in the number of comorbid conditions associated with obesity further increases the risk of general anesthesia [16, 17]. Breast-conserving surgery (BCS) or lumpectomy has the advantage of requiring less time in the operating room and therefore less time requiring anesthesia compared to mastectomy. Following BCS, patients receive radiation therapy to the breast and, depending on their stage, may receive regional nodal radiation as well. While some series identify comparable local control rates in obese and normal-weight women undergoing lumpectomy and radiation therapy, others suggest that local recurrence is significantly higher in obese patients [18, 19•, 20]. What is clearer is that the cosmetic outcome with breast conservation (lumpectomy and radiation) may not be as good in obese women compared to normal-weight women. Serial assessments of breast cosmesis following lumpectomy and radiation therapy have identified persistent changes in the appearance of the breast. Two years after radiation, 31.1% of women with large breasts have documented adverse changes in the breast compared to women with medium-sized (16.3%) and small-sized breasts (4.8%) [21]. Even though more favorable cosmetic outcomes have generally been reported for BCS than mastectomy, as many as 40% of all patients who underwent BCS were dissatisfied with the asymmetry and deformity and these outcomes deteriorate over time [22, 23]. While BCS is associated with a positive body image [24], patients with a BMI > 30 kg/m^2^ were noted to have more postoperative breast asymmetry and less favorable esthetic results [25, 26].

### Mastectomy

While small series from individual cancer centers have reported comparable rates of surgical complications in obese and normal-weight women undergoing mastectomy, larger series which include multiple institutions suggest otherwise. Recent data from the ACS-NSQIP database of 7202 women found that obesity was associated with an increase in both minor and major surgical complications, specifically increased risk of bleeding complications and surgical site infections [27].

### Sentinel Node Mapping

Surgical staging of the axilla is determined by sentinel node mapping in which the primary lymph nodes involved in lymphatic drainage of the breast are removed in order to determine if breast cancer has spread beyond the breast. This procedure has significantly reduced the number of women requiring axillary lymph node dissections, thereby decreasing the incidence of lymphedema [28–30]. However, data from large multi-institutional studies indicate that sentinel lymph node (SLN) identification rates are lower in obese women, and are associated with a higher failure to map rate [31–34].

### Reconstruction

With the rise in both rates of obesity and breast cancer, more obese women are seeking reconstruction following mastectomy. Obese women may not be candidates for reconstruction due to limited reconstructive options or due to comorbidities. A recent systematic review of 29 studies demonstrated that obese women were significantly more likely to have surgical and medical complications following reconstruction compared to normal-weight women. The rate of surgical complications was 2.3 times higher than in non-obese patients, with wound dehiscence being 2.5 times more likely in obese patients. Additionally, obese patients were 2.8 times more likely to have medical complications and have 1.9 times higher risk of reoperation [35•]. Complication rates are also higher in autologous-based (flap) reconstructions in obese patients and include flap failure (both partial and complete), hematomas, necrosis, donor site infections, delayed healing of donor site, seromas, and hernias [36–39]. A separate systematic review evaluating the risk of complications in obese women undergoing breast reconstruction identified that a BMI of 40 was a threshold at which the rate of complications became prohibitively high [39]. Breast reconstruction is ultimately an elective procedure and the high risk of complications in this population may make surgery inadvisable. Additionally, obese patients undergoing breast reconstruction are more likely to be disappointed with the esthetic results [40].

## Radiotherapy

Patients with large breasts may receive increased doses of radiotherapy to critical organs such as the heart or lungs owing to the positioning of the breasts on the chest wall when the patient lies supine [41]. One way to minimize the toxicities associated with radiotherapy in patients with higher BMI and/or large breasts is to use prone whole-breast radiation, which has been found to result in favorable toxicity profiles [42]. Additionally, hypo-fractionated radiotherapy is possible in patients with large breast volumes; however, moist desquamation was found to be four times higher in patients with large breasts compared to those with smaller breasts [43].

## Lymphedema

Lymphedema (LE) is a dreaded complication of treatment for breast cancer. There are known risk factors associated with of the development of LE including the number of lymph nodes removed during surgery, development of surgical complications such as infection or seroma, use of chemotherapy, radiation therapy, and comorbid medical conditions, including obesity. In a recent study, lymphedema rates were also found to be higher in patients undergoing axillary lymph node dissection, and among those with a more advanced stage of disease, in addition to having a higher BMI [12, 14, 32]. Additionally, not only are obese women at greater risk for developing post-operative lymphedema, but obese women are also at a higher risk of pre-operative lymphedema [44•, 45].

## Systemic Therapy

In the adjuvant setting, full doses of chemotherapy are associated with a greater improvement in overall survival [46]. The appropriate dosing of cytotoxic agents for obese women is challenging and underdosing may impact disease outcomes in obese women. Recently, the American Society of Clinical Oncology published guidelines for the “Appropriate Chemotherapy Dosing for Obese Adult Patients With Cancer” [47]. These guidelines were based upon a systematic review in adult survivors with breast, ovarian, colon, or lung cancer, and suggested that reduced doses of chemotherapy may result in poorer disease-free and overall survival. Importantly, there is no clear evidence that short- or long-term toxicities from chemotherapy are increased in obese breast cancer patients receiving full-weight–based doses. The use of fixed-dose chemotherapy is only applicable for certain chemotherapeutic agents (e.g., carboplatin and bleomycin) [47]. Obese patients require higher doses of chemotherapy to achieve therapeutic levels of tumor suppression [48•, 49]. Ewertz et al. reported the 30-year follow-up of 53,816 women enrolled in clinical trials within the Danish Breast Cancer Cooperative Group (DBCCG), of which 18,979 patients had information about BMI [18]. In this study, obesity was associated with an increased risk for developing distant metastatic disease and of dying of breast cancer. Furthermore, the benefits of adjuvant chemotherapy and/or endocrine therapy were significantly less in the obese population, even among women who received appropriate doses of chemotherapy. These findings were independent of tumor size, nodal status, and known prognostic factors, including HR status of the primary tumor [50].

The therapeutic dose of individual endocrine agents is fixed, regardless of weight or body surface area. The Austrian Breast and Colorectal Cancer Study Group (ABCSG) 12 trial randomized 1804 premenopausal women with early-stage breast cancer treated with a luteinizing hormone-releasing hormone (LHRH) agonist for ovarian suppression, to receive adjuvant tamoxifen or anastrozole with a second randomization to zoledronic acid or not [51•]. They reported that overweight women assigned anastrozole had a 60% increase risk of disease recurrence (HR, 1.60; 95% CI, 1.06 to 2.41; *P* = 0.02) and death (HR, 2.14; 95% CI, 1.17 to 3.92; *P* = 0.01), compared to normal-weight survivors. Importantly, the benefits of adjuvant chemotherapy and/or endocrine therapy were significantly less in the obese population and were independent of tumor size, nodal status, and known prognostic factors, including hormone receptor status.

In addition to the DBCCG study, other smaller series have reported less benefit from endocrine therapy in the obese population, independent of tumor size, nodal status, and known prognostic factors, including HR status [18]. In the Arimidex, Tamoxifen Alone or in Combination (ATAC) trial, Sestak et al. reported that postmenopausal breast cancer survivors with a high BMI (BMI > 35 kg/m^2^) at baseline had a significantly higher rate of breast cancer recurrence after 100 months follow-up compared to normal BMI survivors (HR, 1.39; 95% CI, 1.07 to 1.61; *P* = 0.01) [52]. Breast cancer survivors who received anastrozole had a 27% lower recurrence rate than women who received tamoxifen, and the benefit of anastrozole was greater in lower BMI survivors. In contrast, the efficacy of anastrozole was significantly (*P* < 0.01) less effective (higher recurrence rate) in postmenopausal breast cancer survivors with a BMI higher than 30 kg/m^2^ (HR, 1.30; 95% CI, 0.91 to 1.85) compared to those women with a BMI lower than 28 kg/m^2^ (HR, 1.29; 95% CI, 0.92 to 1.81). These data collectively suggest a worse prognosis for overweight and obese women treated with endocrine agents and suggest that aromatase inhibitors may be less effective than tamoxifen in this population [52].

## Biological Factors

Fat is a metabolically active tissue with high levels of the aromatase enzyme which converts androgen to estrogen. Excess estrogen production from expanded adipose tissue has been proposed as a possible mechanism for the adverse outcomes in obese women with breast cancer. However, obesity is associated with adverse disease outcomes in obese women with hormone-sensitive and hormone-resistant cancers [18]. Furthermore, obesity is a risk factor for developing triple-negative breast cancer, which suggests that higher endogenous estrogens may not be the only mechanism contributing to a higher risk of recurrence [53]. Obesity produces inflammation in adipose tissue, and activated macrophages in adipose tissues of obese individuals produce pro-inflammatory mediators such as TNFα and IL-6. Dysregulated inflammation in adipose tissues results in an accumulation of pro-inflammatory T cells and reduction in T regs, which contributes to obesity-related insulin resistance. STAT3 activity is increased in visceral adipose tissues and ablation of STAT3 in T cells has been shown to improve insulin sensitivity and glucose tolerance and reduce inflammation in visceral adipose tissues [54]. JAK2/STAT3 is a regulator of lipid metabolism and promotes breast cancer cell “stemness” and chemoresistance [55]. The biologic basis for the differences in the natural history of breast cancer in obese women is not completely clear. However, biologically active adipose tissues appear to be a contributing factor to the unique pathophysiology in obese women with breast cancer.

## Conclusion

Obese women with breast cancer represent a unique patient population. They are at increased risk for the development of breast cancer and may experience more complications related to surgery and radiation. Despite appropriate local disease treatment, obese women are at increased risk for local recurrence compared to normal-weight women. Similarly, systemic chemotherapy appears to be less effective, even when dosed appropriately on the basis of actual weight. In addition, endocrine therapy may be less effective in obese women, and there is a suggestion that tamoxifen may be more effective than the aromatase inhibitors in this population. Taken together, these data suggest a unique and aggressive biology that is likely due to a tumor environment metabolically activated by adipose tissues. Overall, it is clear that the efficacy of cancer treatments is significantly lower in obese breast cancer survivors, posing greater challenges in patient care and disease management in this patient population. Based upon these challenges, further investigations are warranted to assess the effective diagnostic and treatment mechanisms needed to successfully target breast cancer within the obese patient population.
